# Yi Qi Qing Re Gao formula ameliorates puromycin aminonucleoside-induced nephrosis by suppressing inflammation and apoptosis

**DOI:** 10.1186/s12906-015-0673-9

**Published:** 2015-05-27

**Authors:** Yumin Wen, Yongli Zhan, Huijie Liu, Tingting Zhao, Liping Yang, Haojun Zhang, Xi Dong, Ping Li

**Affiliations:** Beijing University of Chinese Medicine, Beijing, 10029 China; Institute of Clinical Medical Science, China–Japan Friendship Hospital, Beijing, 10029 China; Beijing Key Lab for Immune-Mediated Inflammatory Diseases, Beijing, 10029 China; Department of Nephrology, Guang’anmen Hospital of China Academy of Traditional Chinese Medical Sciences, Beijing, 100053 China; Department of Nephrology, Shunyi Hospital of Traditional Chinese Medicine, Beijing, 101300 China

**Keywords:** Apoptosis, Inflammation, Puromycin aminonucleoside nephrosis, Tumor necrosis factor, YQQRG formula

## Abstract

**Background:**

Yi Qi Qing Re Gao (YQQRG) formula is a traditional Chinese herbal medicine used to treat chronic nephritis. This study was designed to evaluate the underlying mechanism in the use of YQQRG formula to treat nephrosis induced by puromycin aminonucleoside (PAN).

**Methods:**

Thirty-six male Wistar rats were randomly divided into 3 groups of 12 rats each: a sham group, a vehicle-treated PAN model group (PAN), and a group treated with YQQRG (PAN + YQQRG). The PAN model was established by a single intravenous injection of PAN at a dose of 40 mg/kg body weight; rats in the sham group received the same volume of saline. Twenty-four hour urinary protein was measured 0, 3, 5, 10, and 15 days after the injection. The rats were sacrificed on day 10 and day 15 and the serum lipid profile examined. The renal cortex of each rat was stained with periodic acid–Schiff reagent and the pathologic alterations and ultrastructural changes were examined by transmission electron microscopy. *In situ* cell apoptosis was detected by a terminal deoxynucleotidyl transferase-mediated uridine 5′-triphosphate-biotin nick end-labeling (TUNEL) assay. Transcriptive levels of inflammatory markers and molecules associated with apoptosis were detected by a real-time polymerase chain reaction and expression of proteins was examined by either immunohistochemistry or Western blot analysis.

**Results:**

YQQRG significantly decreased urinary protein level, and lowered serum lipid level. YQQRG also attenuated histologic lesions in the rat kidneys. Activation of inflammatory markers was largely restored by the administration of YQQRG. TUNEL assay showed that YQQRG decreased the number of apoptotic cells. Both mRNA and protein levels of caspase-3 were significantly reduced in the group treated with YQQRG, whereas expression of the Bcl-2 protein increased in the YQQRG group.

**Conclusions:**

YQQRG alleviated kidney injury in PAN-treated rats, possibly through anti-inflammatory and anti-apoptotic effects.

## Background

Inflammation and apoptosis are typical pathological features shared by a variety of kidney diseases, such as glomerulosclerosis and tubulointerstitial fibrosis, which consequentially induce renal failure. Prevention and treatment of renal inflammation or apoptosis may be a key issue in treating kidney disease.

There is a growing body of evidence indicating that inflammation is a crucial contributor to the pathogenesis of most kidney diseases [1]. The main mechanism of inflammation in kidney damage is as follows [2]. Chemokines or adhesion molecules secreted by resident renal cells attract circulating inflammatory cells and induce infiltration of the inflammatory cells. Activated inflammatory cells, such as macrophages and neutrophils, are responsible for the production of various proinflammatory mediators. Proinflammatory cytokines have several biologic effects, including direct cytotoxicity toward renal cells, increased synthesis of other inflammatory molecules and the triggering of cell apoptosis. Various macrophage inhibition strategies have been used to successfully treat kidney diseases in animal models [3]. Monocyte chemoattractant protein (MCP-1), which triggers macrophage recruitment, has been found to be secreted by tubular epithelial cells in both patients with proteinuria and in proteinuric animal models.

Apoptosis is essential in the development of mammalian kidneys and in the removal of excess glomerular cells in the resolution of proliferative nephritis. However, severe apoptosis caused by environmental and intrinsic stimuli results in a loss of resident kidney cells and, thus consequently, kidney injury [4]. Inflammation and apoptosis are closely linked. Macrophages in the kidney are responsible for apoptotic cell death induced by puromycin aminonucleoside (PAN). Cytokine tumor necrosis factor α (TNF-α) is a prominent stimulator of apoptosis. In chronic proteinuric renal disease, inflammation and apoptosis are implicated in glomerular sclerosis and tubular atrophy [5].

Yi Qi Qing Re Gao (YQQRG) formula, a traditional Chinese herbal medicine, has been used to treat chronic nephritis for over 2 decades in Guang’anmen Hospital, Beijing. Our previous studies have found that YQQRG decreases the excretion of urinary protein in patients with chronic nephritic and in the rat model of adriamycin-induced nephrosis [6, 7]. Treatment with serum containing YQQRG, which was collected from YQQRG treated healthy Wistar rats, ameliorated lipopolysaccharide-stimulated rat mesangial cell proliferation in vitro (L Yang, et al. unpublished data). Another study found that pretreatment with YQQRG could protect the filtration barrier from PAN-induced architectural damage [8]. Since lipopolysaccharide is a powerful stimulus for inflammation, and apoptosis is frequently involved in architectural damage, we hypothesize the renoprotective effects of YQQRG may due to its anti-inflammatory and anti-apoptotic actions. The study reported here was designed to identify the anti-inflammatory and anti-apoptotic effects of YQQRG on nephrosis induced by PAN.

## Methods

### Herbal formulation and reagents

YQQRG formula is a natural herbal medicine formulated based on the empirical experience of a Chinese medicine expert. The ingredients of YQQRG formula and proportions for each herb are: *Astragalus membranaceus* (Fisch.) Bge 6.2 %, *Atractylodes macrocephala* Koidz 4.6 %, *Saposhnikovia divaricata* (Turcz.) Schischk 3.1 %, *Lonicera japonica* Thunb 6.2 %, *Forsythia suspensa* (Thunb.) Vahl 6.2 %, *Duchesnea indica* (Jacks.) Focke 4.6 %, *Oldenlandia diffusa* (Willd.) Roxb 15.4 %, *Poria cocos* (Schw.) Wolf 6.2 %, *Alisma plantago-aquatica* L 10.7 %, *Leonurus japonicus* Houtt 15.4 %, *Imperata cylindrica* (L.) P. Beauv 15.4 %, and *Dioscorea nipponica* Makino 6.2 %. All herbs were purchased from Kangmei Pharmaceutical (Beijing, China). The herbs were combined and decocted with water. The decoction was then concentrated, ethanol precipitated, and finally sterilized. The resulting preparation was creamy with each milliliter equaling 4.5 g herbs. PAN and podocin antibody were purchased from Sigma-Aldrich (St. Louis, MO, USA). TNF-α, MCP-1, interleukin-1 beta (IL-1β) and CD68 antibodies were obtained from Santa Cruz Biotech (Santa Cruz, CA, USA). TNFR1 antibody was purchased from Abcam (Cambridge, MA, USA). iNOS and cleaved caspase-3 antibodies were purchased from Cell Signaling Technology (Beverly, MA, USA). Bcl-2 rabbit polyclonal antibody was purchased from Bioworld Technology (St. Louis Park, MN, USA). β-actin mouse monoclonal antibody, affinity-purified anti-mouse antibody and anti-rabbit antibody were obtained from Jackson ImmunoResearch (West Grove, PA, USA). A terminal deoxynucleotidyl transferase-mediated uridine 5′-triphosphate-biotin nick end-labeling (TUNEL) kit was purchased from Roche Diagnostics GmbH (Penzberg, Germany).

### Animals

Male Wistar rats weighing 90–100 g were purchased from Beijing HFK Bio-Technology (Beijing, Certificate No. SCXK 2009–0007). After 3 days of adaptive feeding, the animals were randomly divided into 3 groups of 12 rats each: a sham group; a vehicle-treated PAN model group (PAN); and an YQQRG treatment group (PAN + YQQRG). YQQRG was administered daily to the 12 YQQRG-treated rats by gavage at a dosage of 4 g/kg body weight per day for 1 week before PAN injection and 15 days after PAN injection. The 12 PAN model animals received distilled water. The PAN model was established by a single intravenous injection of PAN at a dose of 40 mg/kg body weight. PAN was freshly dissolved in normal saline at a concentration of 1.33 mg/ml. The left internal jugular vein was isolated and was ligated at the proximal end with an intraperitoneal injection of 10 % chloral hydrate (1 ml/300 g body weight). The jugular vein was then punctured with an intravenous cannula connected to a syringe filled with 3 ml of PAN solution. The solution was injected over 5 min. The jugular vein was ligated after the injection. The sham group received a single injection of 3 ml of normal saline. All animals were housed in a temperature-controlled environment at 22 ± 3 °C and 50 ± 10 % humidity under a 12-h light/dark cycle and were allowed free access to tap water and standard chow. The study protocol was approved by the Ethics Committee of the China–Japan Friendship Hospital and was performed in accordance with the Guiding Principles for the Care and Use of Laboratory Animals.

### Serum and urine analysis

To determine urinary protein level, rats were placed into metabolic cages 0, 3, 5, 10 and 15 days after injection of PAN. Twenty-four hour urine samples were collected in brown bottles and total urinary protein was measured using the Bradford method [9]. Six rats in each group were randomly sacrificed 10 and 15 days after the injection of PAN. Blood samples were collected from their abdominal aorta for the determination of triglycerides (TG) and low-density lipoprotein-cholesterol (LDL-C) levels using an automatic biochemistry analyzer (Bayer, Berlin, Germany).

### Histologic examination

Both kidneys were removed after collection of the blood sample. For examination by transmission electron microscopy, the cortex from the upper pole of the right kidney was cut into 1 mm^3^ sections, fixed by immersion in 2.5 % glutaraldehyde, dehydrated with gradient acetone and embedded with Epon 812 ethoxyline resin. The samples were subsequently prepared as ultra-thin serial sections and examined with a transmission electron microscope (H-600, Hitachi, Tokyo, Japan). For the light microscopy examination, the kidneys were cut into transverse sections and fixed in 10 % phosphate-buffered formalin solution (pH 7.4) for over 24 h. After fixation, the sections were embedded in paraffin and prepared as 3 μm slides for periodic acid–Schiff (PAS) staining. Results were observed using a BX-51 Research Microscope System and a DP70 Image Acquisition System (Olympus, Tokyo, Japan).

### Real-time polymerase chain reaction

Total RNA was extracted from the kidney cortex of each rat using TRIzol reagent (Invitrogen, Carlsbad CA, USA). A total of 3 μg of RNA from each sample was reverse-transcripted using a cDNA synthesis kit (Thermo Fisher Scientific, Vilnius, Lithuania) following the manufacturer’s instructions. The target genes were amplified in an ABI 7500 real-time PCR system (Life Technologies, Grand Island, NY, USA). Reactions were carried out in 96-well plates with 25 μl volumes containing 30 ng of cDNA, 200 nM of each primer and 12.5 μl 2× SYBR Green Master Mix Reagent. Each reaction was performed in triplicate. ß-Actin was used as an internal control. The primers were generated using Oligo 7 software (Molecular Biology Insights, Colorado Springs, CO, USA) (Table 1). Data were analyzed by the 2^−ΔΔ*CT*^ method [10].

**Table 1:** Primers for real-time-PCR

	Primer	Sequence
TNF-α	Forward	GCCCAGACCCTCACACTC
	Reverse	TCCTGGTATGAAATGGCAAA
TNFR1	Forward	TTTACAGCTTCGCAGAACCAC
	Reverse	CACACACTGGAAATGCGTCT
IL-1β	Forward	AGCAGCATCTCGACAAGAGC
	Reverse	ATCACACACTAGCAGGTCGTC
MCP-1	Forward	CTGCTGCTACTCATTCACT
	Reverse	CCATTCCTTATTGGGGTCA
Caspase-3	Forward	AGCAGTTACAAAATGGATTAC
	Reverse	ATCTCCATGACTTAGAATCAC
Bcl-2	Forward	TGAGTACCTGAACCGGCATC
	Reverse	AGAAATCAAACAGAGGTCGCAT
β-actin	Forward	ACCCTAAGGCCAACCGTGAAAAG
	Reverse	CATGAGGTAGTCTGTCAGGT

### Immunohistochemistry

Paraffin sections were prepared by dewaxing and rehydrating. Endogenous peroxidase activity was suppressed by incubating the section in 3 % H_2_O_2_ for 10 min. The antigens were then demasked by heat-induced epitope retrieval in either pH 6 citrate buffer or pH 9 Tris–EDTA buffer according to the manufacturer’s instructions for each antibody. The sections were incubated overnight at 4 °C with primary antibodies diluted in antibody dilution buffer. After gently washing with Tris-buffered saline (TBS), the sections were incubated with horseradish peroxidase enzyme labeled secondary antibodies at 4 °C for 30 min. The reaction was visualized by adding DAB substrate solution and counterstaining with hematoxylin. Image-Pro Plus 6.0 image analysis software (Media Cybernetics, Warrendale, PA, USA) was used to semi-quantify the results.

### TUNEL assay

Apoptosis was detected *in situ* using a TUNEL kit according to the manufacturer’s instructions. After dewaxing and rehydrating, paraffin sections were treated with protease (20 μg/ml) for 30 min at 37 °C and then incubated with 50 μl of TUNEL reaction mixture for 30 min at 37 °C in the dark. This was followed by treatment with 50 μl of converter-POD solution for 30 min at 37 °C and incubation with DAB substrate solution before analysis by light microscopy. The nuclei of apoptotic cells were stained dark brown.

### Western blotting

Kidney cortices were isolated and frozen in liquid nitrogen. A total of 40 μg protein per lane was separated on 12 % SDS-PAGE gel and electrophoretically transferred to PVDF membranes. The membranes were incubated overnight at 4 °C with the relevant primary antibodies, followed by incubation with appropriate secondary antibodies at room temperature for 1 h. After washing 3 times with TBS, the bands were visualized by ECL Western blotting detection reagents (Engreen Biosystem, Beijing, China) and analyzed semi-quantitatively by the Image J software (National Institutes of Health, Bethesda, MD, USA).

### Statistical analysis

Results are expressed as mean ± SD. Multiple comparisons among groups were performed using one-way analysis of variance followed by the Student–Newman–Keuls test. Differences with *P* < 0.05 were considered significant.

## Results

### Effect of YQQRG proteinuria and serum lipid disorder

**Fig. 1:**
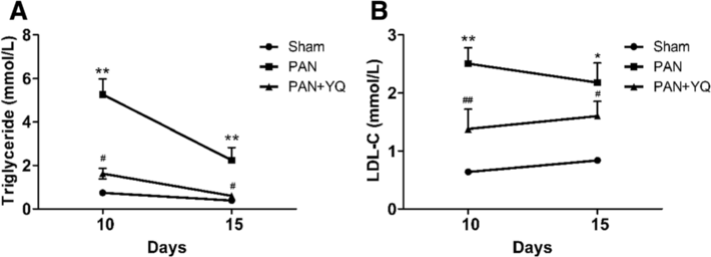
Serum lipid changes at indicated time points after the injection of PAN. **A** Triglyceride levels. **B** LDL-C levels. Rats in the PAN + YQ group received a daily oral gavage of YQQRG. Rats in the sham and PAN groups received the same volume of vehicle (distilled water). ^*^ *P* < 0.05 and ^**^ *P* < 0.01 compared with the sham group; ^#^ *P* < 0.05, ^*##*^ *P* < 0.01 compared with the PAN model group. LDL-C, low-density lipoprotein-cholesterol

As shown in Table 2, before the injection of PAN, the total urinary protein in each group was below 2 mg/24 h. Proteinuria level in the PAN group was increased to 110.4 mg/24 h five days after the injection of PAN (*P* < 0.01). Proteinuria level in the PAN group peaked at 338.2 mg/24 h on day 10 (*P* < 0.01) and decreased to 226.84 mg/24 h a day 15 (*P* < 0.01), whereas Proteinuria level in the sham group remained unchanged. Treatment with YQQRG significantly decreased Proteinuria level on days 10 and 15 (*P* < 0.05). In addition, the PAN group exhibited an increase in TG and LDL-C levels, which are typical manifestations of nephrotic syndrome. YQQRG persistently decreased the levels of TG and LDL-C (Fig. 1).

**Table 2:** Effects of YQQRG on urinary protein levels (mg/24 h)

	Day 0	Day 3	Day 5	Day 10	Day 15
Sham	1.01 ± 0.16	1.38 ± 0.18	2.89 ± 1.19	5.32 ± 1.70	6.52 ± 2.55
PAN	1.25 ± 0.60	2.36 ± 0.56	110.40 ± 48.85**	338.20 ± 93.24**	226.84 ± 87.63**
PAN + YQQRG	1.26 ± 0.51	2.99 ± 1.31	134.85 ± 57.13**	238.02 ± 55.78**^##^	170.87 ± 35.77**^#^

Data are presented as mean ± SD. ^**^ *P* < 0.01 compared with sham group. ^#^ *P* < 0.05, ^##^ *P* < 0.01 compared with PAN group

### Influences of YQQRG renal morphology

**Fig. 2:**
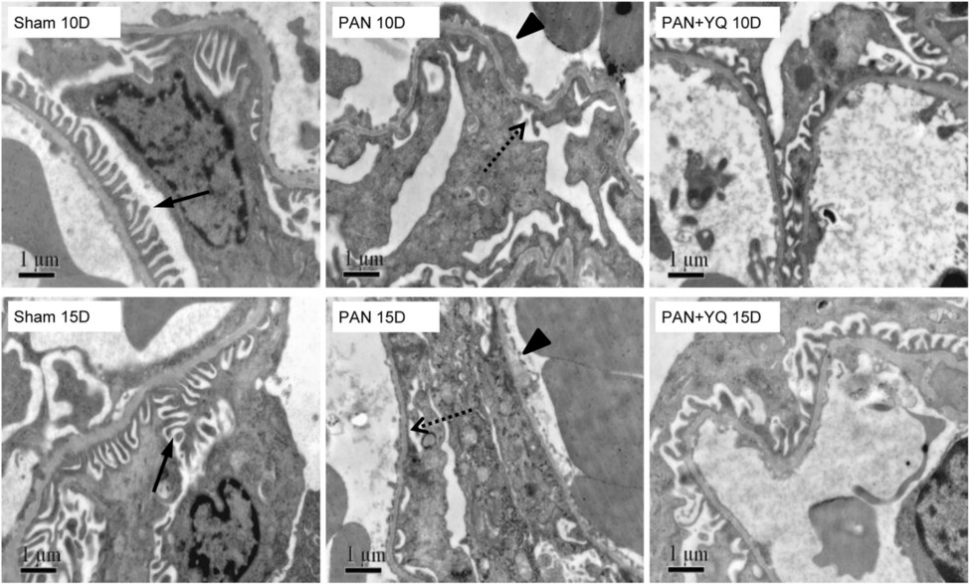
Ultrastructural measurement of glomeruli by electron microscopy. Rats in the PAN + YQ group received a daily oral gavage of YQQRG. Rats in the sham and PNA groups received the same volume of vehicle (distilled water). 10D, 10 days after PAN injection; 15D, 15 days after PAN injection; back arrow, podocyte foot process in the sham panel; dotted arrow, diffuse foot process effacement in PAN panel; arrow head, segmental endothelial cell swelling in PAN panel

**Fig. 3:**
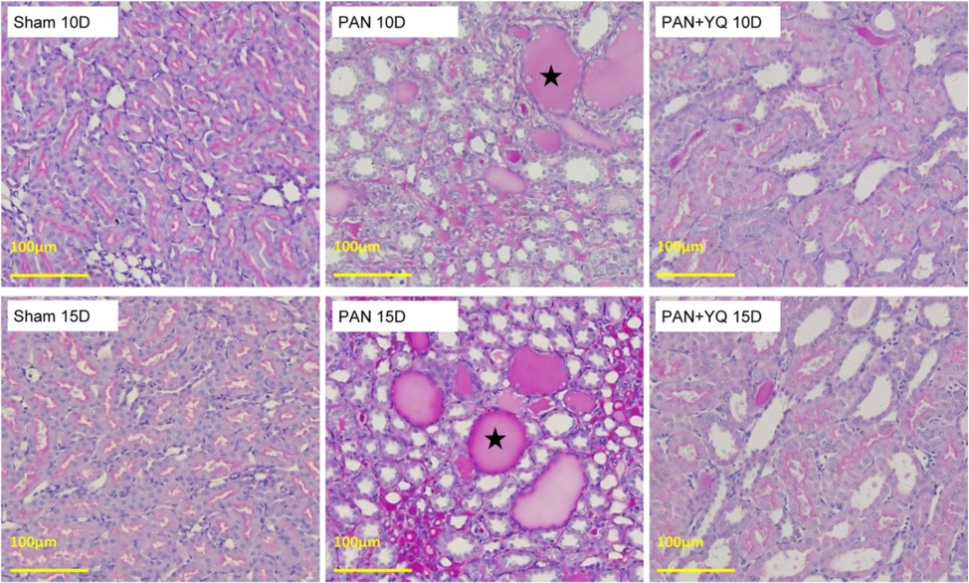
Representative morphological changes in tubules (periodic acid–Schiff staining). Rats in the PAN + YQ group received a daily oral gavage of YQQRG. Rats in the sham or PNA groups received the same volume of vehicle (distilled water). 10D, 10 days after PAN injection; 15D, 15 days after PAN injection. The protein cast in the distal tubules and collecting tubules is indicated by a black star in the PAN panel

Transmission electron microscopy revealed obvious architectural abnormalities of the filtration barrier in the PAN group (Fig. 2). The sham group presented a normal structure with a thin and regular arrangement of the foot processes. However, diffuse podocyte foot process effacement along with prominent endothelial cell swelling were observed in the PAN group at each time point. Treatment with YQQRG markedly reduced the severity of foot process effacement compared with the PAN group. PAS staining did not reveal abnormal glomerular structures in any of the groups (data not shown). In parallel with the severity of proteinuria, the PAN group showed persistent protein casts in the distal tubules and collecting tubules on days 10 and 15, whereas the sham group exhibited a normal structure (Fig. 3). Treatment with YQQRG restored this change.

### Effect of YQQRG on podocyte injury

**Fig. 4:**
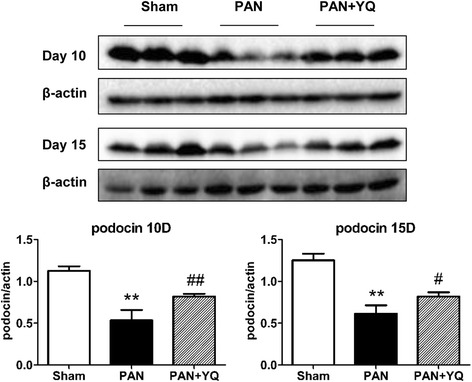
Effect of YQQRG on podocin expression. Podocin expression is evaluated by Western blot. The upper panel indicates the original band observed by Western blot. The lower panel displays semi-quantitative results. Rats in the PAN + YQ group received a daily oral gavage of YQQRG. Rats in the sham and PNA groups received the same volume of vehicle (distilled water). 10D, 10 days after PAN injection; 15D, 15 days after PAN injection. Data are presented as mean ± SD values for each group of 3 animals. ^**^ *P* < 0.01 compared to sham group, respectively. ^#^ *P* < 0.05 and ^##^ *P* < 0.01 compared with PAN group, respectively.

To further verify the successful establishment of the PAN model in this study and observe the effect of YQQRG on podocyte injury, the expression of podocin was examined by Western Blot. Podocin is specifically localized to the podocyte foot processes, which function to maintain the glomerular filtration permeability. In PAN induced nephrosis, podocin expression is markedly decreased due to the severe podocyte injury [11]. Podocin expression was largely decreased in the PAN model group at both time points, while it was upregulated by YQQRG treatment (Fig. 4).

### Action of YQQRG on the activation of inflammatory markers in the kidney

**Fig. 5:**
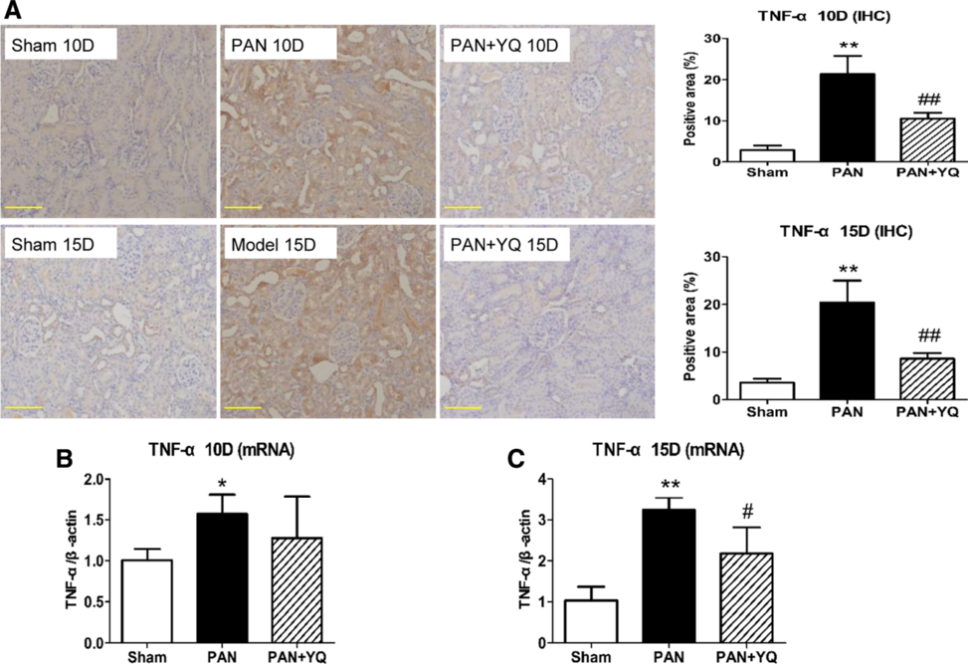
Effect of YQQRG treatment on expression of TNF-α in the kidney. Expression of TNF-α protein was determined by immunohistochemistry staining and relative semi-quantified assay (**A**). Transcription level of TNF-α was examined by real-time PCR at 10 days (**B**) and 15 days (**C**) after injection of PAN. Rats in the PAN + YQ group received a daily oral gavage of YQQRG. Rats in the sham and PNA groups received the same volume of vehicle (distilled water). 10D, 10 days after PAN injection; 15D, 15 days after PAN injection. Data are presented as mean ± SD values for each group of 3 animals. *P < 0.05 and ^**^ *P* < 0.01 compared to sham group, respectively. ^#^ *P* < 0.05 and ^##^ *P* < 0.01 compared with PAN group, respectively. (magnification × 200, scale bar = 100 μm)

**Fig. 6:**
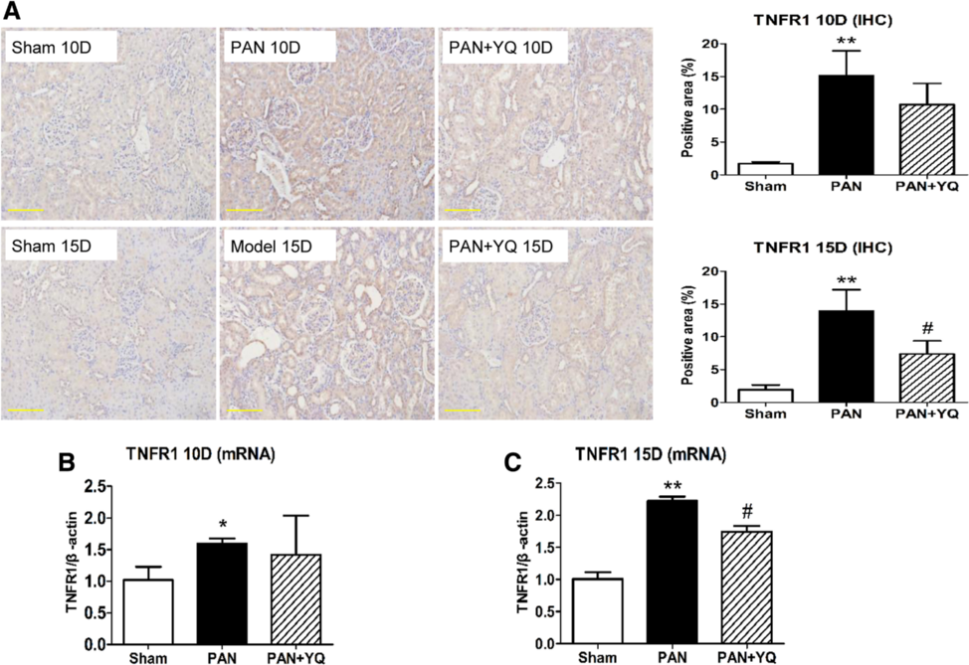
Effect of YQQRG treatment on renal expression of TNFR1. TNFR1 was detected by immunohistochemistry (**A**) and real-time PCR (**B** and **C**). Rats in the PAN + YQ group received a daily oral gavage of YQQRG. Rats in the sham and PNA groups received the same volume of vehicle (distilled water). 10D, 10 days after PAN injection; 15D, 15 days after PAN injection. Data are presented as mean ± SD values for each group of 3 animals. ^*^ *P* < 0.05 and ^**^ *P* < 0.01 compared with the sham group. ^#^ *P* < 0.05 compared with PAN group, respectively. (magnification × 200, scale bar = 100 μm)

**Fig. 7:**
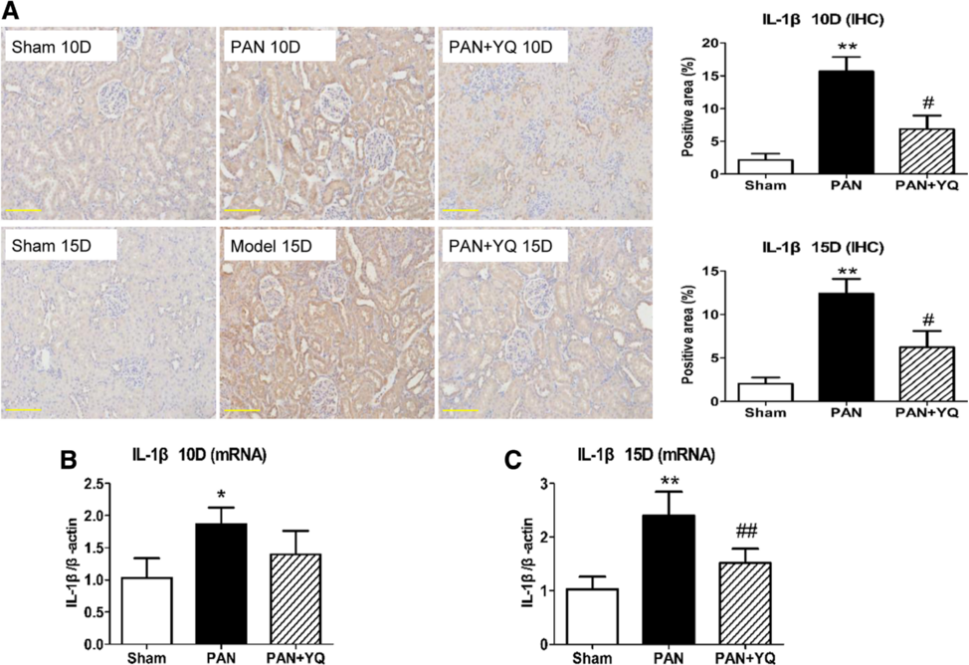
Effect of YQQRG treatment on renal expression of IL-1β. The inflammatory cytokine IL-1β was observed by immunohistochemistry assay (**A**), along with its gene transcription examined by real-time PCR in the renal cortex (**B** and **C**). Rats in the PAN + YQ group received daily oral gavage of YQQRG. Rats in the sham and PNA groups received the same volume of vehicle (distilled water). 10D, 10 days after PAN injection; 15D, 15 days after PAN injection. Data are presented as mean ± SD values for each group of 3 animals. ^*^ *P* < 0.05 and ^**^ *P* < 0.01 compared to sham group, respectively. ^#^ *P* < 0.05 and ^##^ *P* < 0.01 compared to PAN group, respectively. (magnification × 200, scale bar = 100 μm)

**Fig. 8:**
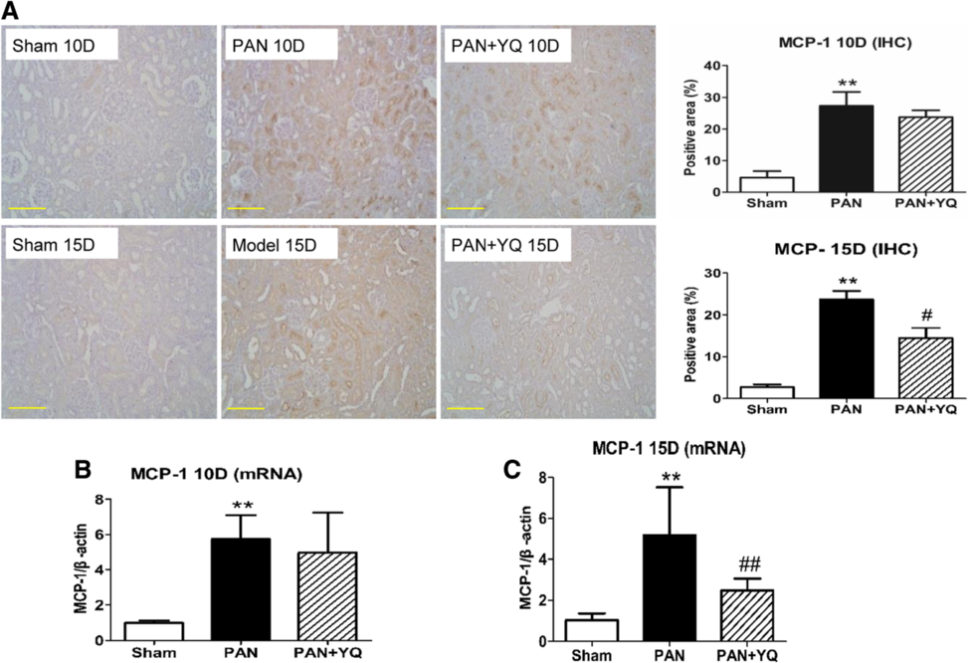
Effect of YQQRG treatment on renal expression of MCP-1. The chemokine MCP-1 was assessed by immunohistochemistry (**A**) and real-time PCR (**B** and **C**). Rats in the PAN + YQ group received daily oral gavage of YQQRG. Rats in the sham and PNA groups received the same volume of vehicle (distilled water). 10D, 10 days after PAN injection; 15D, 15 days after PAN injection. Data are presented as mean ± SD values for each group of three animals. ^**^ *P* < 0.01 compared to sham group, respectively. ^#^ *P* < 0.05 and ^##^ *P* < 0.01 compared to PAN group, respectively. (magnification × 200, scale bar = 100 μm)

**Fig. 9:**
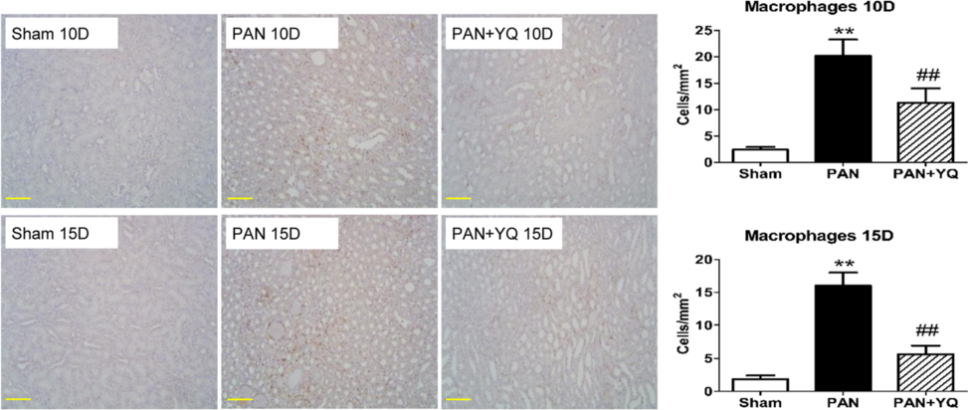
Effect of YQQRG treatment on renal macrophage infiltration. Macrophages were assessed as CD68-positive cells in the kidney. Quantification results are shown for the number of CD68-positive cells. Rats in the PAN + YQ group received daily oral gavage of YQQRG. Rats in the sham and PNA groups received the same volume of vehicle (distilled water). 10D, 10 days after PAN injection; 15D, 15 days after PAN injection. Data are presented as mean ± SD values for each group of three animals. ^**^ *P* < 0.01 compared to sham group, respectively. ^##^ *P* < 0.01 compared to PAN group, respectively. (magnification × 200, scale bar = 100 μm)

To observe the anti-inflammatory effects of treatment with YQQRG, changes in inflammatory mediators were examined in this model. In agreement with previously published reports [12–14], inflammatory markers involved in the PAN model, including cytokine TNF-α (Fig. 5) and its receptor TNFR1 (Fig. 6), IL-1β (Fig. 7) and chemokine MCP-1 (Fig. 8), were detected by immunohistochemistry and real-time PCR. Macrophage infiltration was evaluated by immunohistochemistry staining of CD68 (Fig. 9). These markers of expression were all upregulated at both time points after PAN injection in the PAN group. Most of the mediators were localized to the tubules and interstitium, except for the distribution pattern of TNFR1 in both tubules and glomeruli. Treatment with YQQRG resulted in a persistent statistically significant decrease in TNFα, IL-1β and CD68 after injection with PAN, whereas TNFR1 and MCP-1 were decreased on day 15. In terms of the level of transcription, significant decreases as a result of YQQRG treatment were found on day 15.

### Action of YQQRG on renal iNOS expression

**Fig. 10:**
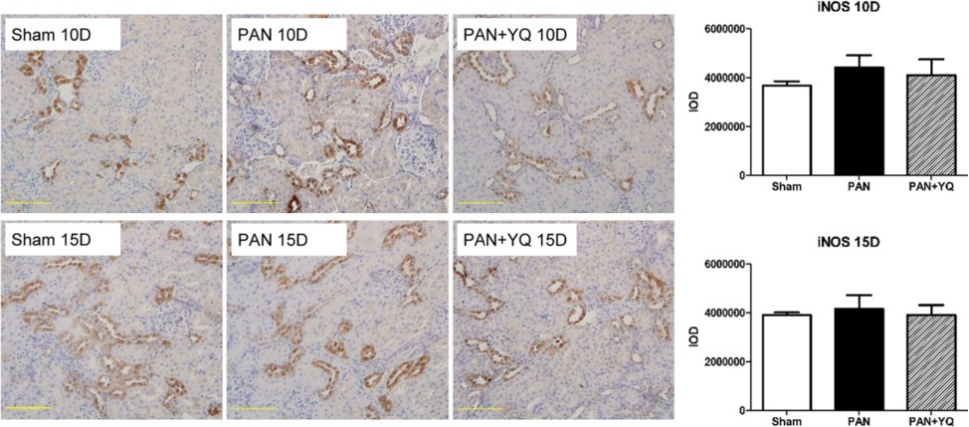
Action of YQQRG on renal iNOS expression. Renal iNOS expression was assessed by immunohistochemistry. Rats in the PAN + YQ group received a daily oral gavage of YQQRG. Rats in the sham and PNA groups received the same volume of vehicle (distilled water). 10D, 10 days after PAN injection; 15D, 15 days after PAN injection. Data are presented as mean ± SD values for each group of 3 animals. (magnification × 200, scale bar = 100 μm)

Inducible nitric oxide Synthase (iNOS) is usually activated by inflammation to produce excessive reactive oxygen species (ROS), which can greatly damage cells. Physiologically, iNOS produces the vasoactive substance nitric oxide (NO), which is essential for blood pressure control and the kidney development. In order to evaluate the action of YQQRG on inflammation induced oxidative stress, this study examine the renal iNOS expression by immunohistochemistry. iNOS expressions in both the PAN group and PAN + YQQRG group did not show statically significant difference with the sham group, although the PAN group exhibit an increasing trend in iNOS expression (Fig. 10).

### Effect of YQQRG on TUNEL-positive apoptosis cells

**Fig. 11:**
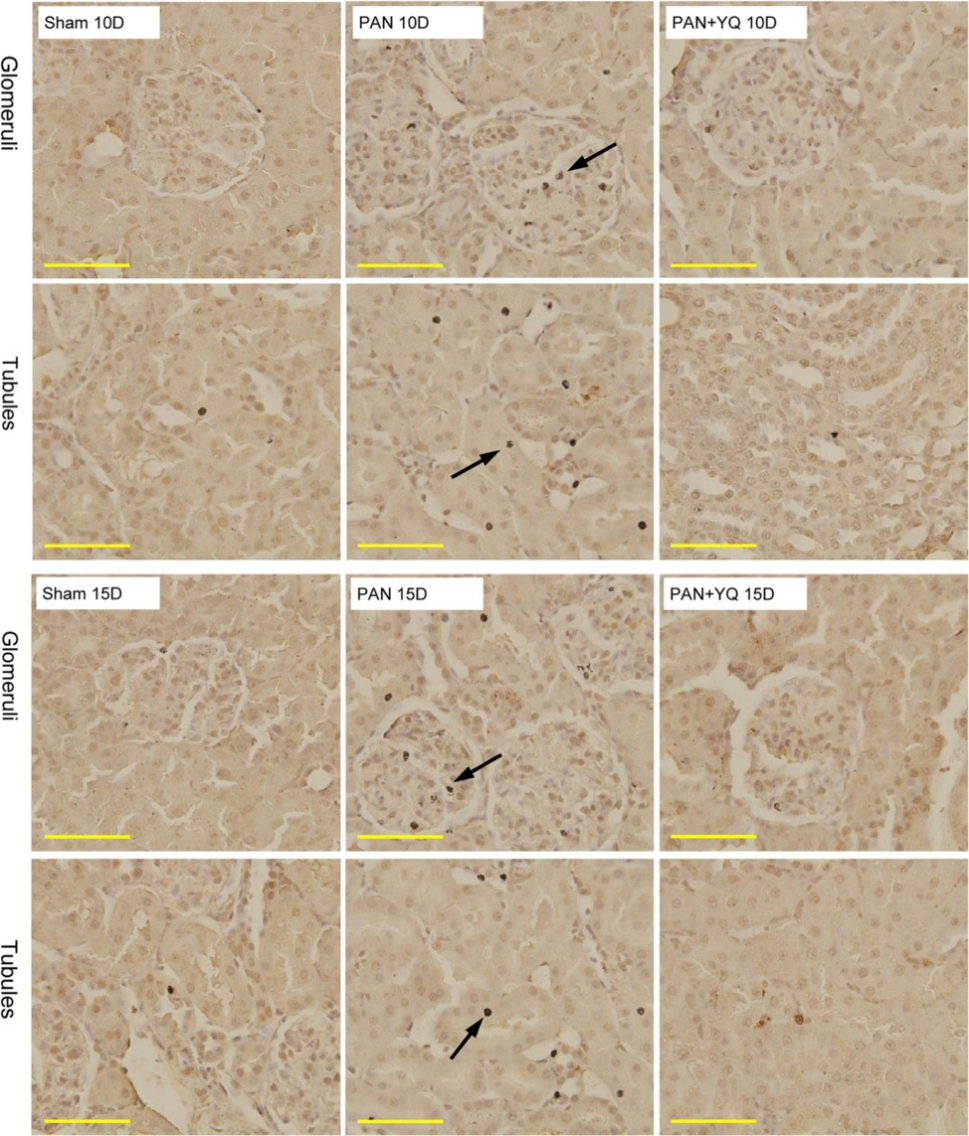
*In situ* cell apoptosis detected by TUNEL assay. Rats in the PAN + YQ group received a daily oral gavage of YQQRG. Rats in the sham and PNA groups received the same volume of vehicle (distilled water). 10D, 10 days after PAN injection; 15D, 15 days after PAN injection. Apoptotic cells (arrow) are illustrated in the PAN panel. (magnification × 400, scale bar = 50 μm)

Apoptotic cells were rarely present in the sham group, whereas the PAN group showed a significant increase in glomerular and tubular apoptosis at each time point (Fig. 11). Histologic observations revealed that the apoptotic cells were resident cells, mainly podocytes and endothelial cells in the glomeruli and epithelial cells in the proximal and distal tubules. Treatment with YQQRG decreased the number of apoptotic cells in both the glomeruli and tubules.

### Effects of treatment with YQQRG on the renal expression of the apoptosis-associated proteins caspase-3 and Bcl-2

**Fig. 12:**
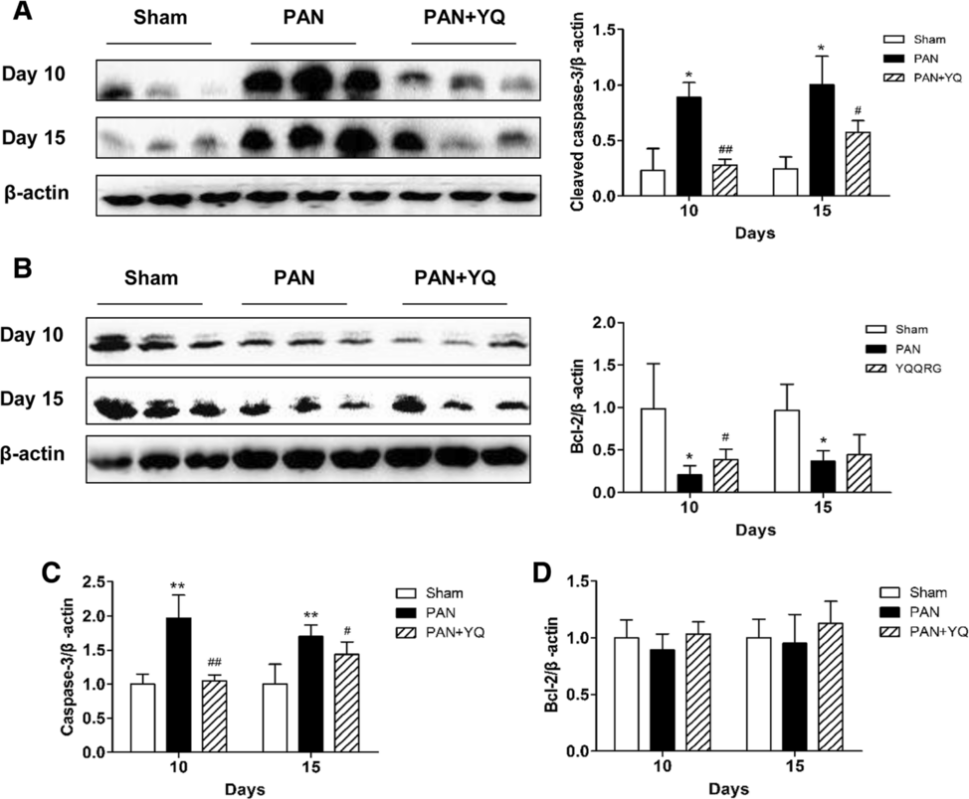
Effect of YQQRG treatment on the apoptosis-associated molecules caspase-3 and Bcl-2 expression. Protein expression of cleaved caspase-3 (**A**) and Bcl-2 (**B**) were examined by Western blot analysis. mRNA expression of caspase-3 (**C**) and Bcl-2 (**D**) were assessed by real-time PCR. Rats in the PAN + YQ group received a daily oral gavage of YQQRG. Rats in the sham and PNA groups received the same volume of vehicle (distilled water). 10D, 10 days after PAN injection; 15D, 15 days after PAN injection. Data are presented as mean ± SD values for each group of three animals.^*^ *P* < 0.05, ^**^ *P* < 0.01 compared with sham group; ^#^ *P* < 0.05, ^##^ *P* < 0.01 compared with PAN group

Western blot quantification showed a potent increase in expression of cleaved caspase-3 protein in the PAN group compared with the sham group (*P* < 0.01). Treatment with YQQRG decreased expression of cleaved caspase-3 at both time points (Fig. 12A). Injection with PAN significantly decreased level of Bcl-2 protein at both time points (*P* < 0.05). Treatment with YQQRG increased its expression, but statistical significance was only found on day 10 (*P* < 0.05) (Fig. 12B). In a similar manner to the expression of protein, real-time-PCR analysis revealed that mRNA expression of caspase-3 increased persistently after injection of PAN in the PAN group compared with the sham group (*P* < 0.01). Treatment with YQQRG significantly decreased its expression on day 10 (*P* < 0.01) and on day 15 (*P* < 0.05) after injection with PAN compared with the model group (Fig 12C). However, mRNA expression of Bcl-2 did not change after injection with PAN (Fig 12D).

## Discussion

PAN-induced nephrosis is a well-established animal model that mimics human minimal change disease, which is characterized by massive proteinuria and hyperlipemia [15]. The acute phase of PAN-induced nephrosis lasts about 2 weeks, after which the kidneys undergo self-healin. The mechanisms of recovery are currently unknown. Typical histologic changes in this model are abnormalities of the glomerular epithelial cells and endothelial cells. Numerous studies have confirmed that nephrotic-range proteinuria is accompanied by kidney lesions, increased inflammation and apoptosis, which indicates that inflammation together with apoptosis may be the underlying mechanism of these changes.

In the present study, we found that treatment with YQQRG significantly decreased 24-h urinary protein levels and improved serum lipid disorder in PAN-induced nephrosis, providing evidence that YQQRG may ameliorate nephrotic syndrome *in vivo*. Morphologic study revealed that treatment with YQQRG could preserve the filtration barrier architecture and decrease the amount of protein cast in correlation with the inhibition of proteinuria. At the same time, YQQRG could restore the expression of podocin in the podocyte. As inflammation and apoptosis promote progression of kidney injuries in this model, we hypothesize that the renoprotective function of YQQRG may be at least partly a result of its anti-inflammatory and anti- apoptotic effects.

As TNF-α is a strong mediator of inflammation, this study focused on the changes in TNF-α and its receptor TNFR1. TNF-α is typically synthesized by monocytes/macrophages, whereas in the kidney various intrinsic cells, including tubular epithelial cells, podocytes, mesangial cells and endothelial cells, could be the original source of TNF-α synthesis. The pathophysiologic role of TNF-α in mediating kidney disease has been well defined by numerous studies. Increased circulatory and urinary levels of TNF-α have been found to be independently associated with increased proteinuria in patients with diabetic nephropathy. Renal TNF-α accumulation is also involved in increasing the permeability of albumin. In accordance with the level of urinary protein, TNF-α production in the kidney is largely increased in the acute phase and gradually decreased 2 weeks after the administration of PAN. TNF-α is directly toxic to intrinsic renal cells and its toxicity can be successfully abolished by anti-TNF antibodies [12]. TNF-α interacts with its receptors TNFR1 and TNFR2 to recruit inflammatory cells and initiate apoptosis. In this study, we examined the signaling regulatory effect of YQQRG on TNF.

A significant positive correlation has been reported in the PAN model between the severity of interstitial nephritis, as determined by increased CD68 positive macrophages, MCP-1 and IL-1β, and the degree of proteinuria. This study showed that YQQRG decreased the infiltration of macrophages in the tubular interstitium. Other inflammatory cytokine and chemokine activations involved in PAN-induced nephrosis, including IL-1β and MCP-1, were abolished by treatment with YQQRG.

The role of iNOS and NO in PAN model remains to be elucidated. There are contradictory reports in the literature on iNOS expression in the PAN model. Most studies have demonstrated that ROS production is upregulated in PNA-treated animals [16, 17]. However, Ni et al. [18] observed that iNOS as well as NO metabolites were decreased in PAN-treated rats, concluding the decreased iNOS was secondary to increased proteinuria. In the current study, immunohistochemistry indicated iNOS was localized to the periglomerular tubules in the kidney cortex, consistent with the finding reported by Walker et al. [19]. We observed that the PAN treated group did not differ from the sham group in iNOS expression, and YQQRG treatment did not influence iNOS expression.

Podocyte apoptosis is largely involved in PAN-induced nephrosis and the progression of focal segmental glomerulosclerosis [20]; PAN causes apoptosis in cultured podocytes [21]. In our previous study, we found that pretreatment with YQQRG inhibited reduction of podocyte-specific molecular nephrin, podocin and CD-2AP in the PAN model [8]. In this study, we found significant cell apoptosis in the podocytes and tubules, which treatment with YQQRG could reduce. Unlike its direct injury to glomeruli, PAN is not toxic towards tubular cells [22]. Apoptosis of the tubular epithelium is probably attributed to the impact of proteinuria and lipid toxicity [23]. Albumin bound to fatty acids in urine is reabsorbed into the proximal tubular epithelial cells and results in apoptosis and tubular damage [24]. As a major characteristic of the PAN nephrosis model, increased serum lipids have been recognized to be a powerful stimulus of cellular apoptosis [25]. Treatment with YQQRG may decrease serum levels of TG and LDL-C. We therefore deduced that the reduction of apoptosis by treatment with YQQRG may be partially from its lipid lowering function. Treatment with YQQRG may inhibit tubular epithelial cell apoptosis by regulating lipid disorders and decreasing urinary excretion of albumin.

To further clarify the mechanism of its anti-apoptotic action, we examined the influence of YQQRG on the expression of two major apoptosis-regulating factors: caspase-3 and Bcl-2. As a major executor of apoptosis, caspase-3 is considered to be the final downstream protein required for apoptosis [26]. Once activated, cleaved caspase-3 mediates cleavage of various substrates to trigger apoptosis. Bcl-2 is a pivotal anti-apoptotic member of the Bcl family. The major contribution of Bcl-2 is in maintaining cellular calcium homeostasis to inhibit activation of calpain and, thus to suppress the cleavage of caspase-4/12 in the outer membrane of the endoplasmic reticulum [27]. In addition, Bcl-2 has the ability to form heterodimers with the pro-apoptotic members of the Bcl family Bax/Bak, resulting in a decrease in pro-apoptotic ability and promotion of cell survival [28]. We observed that YQQRG could downregulate expression of caspase-3 at both the transcriptional and translational levels. Treatment with YQQRG increased expression of Bcl-2 protein. In this way, YQQRG may act as an efficient anti-apoptotic agent.

Several ingredients in YQQRG have anti-inflammatory and anti-apoptotic effects. *Astragalus membranaceus* is the major ingredient of YQQRG and there is abundant evidence that its active component, astragaloside IV, has a prominent anti-inflammatory effect through the inhibition of cytokine expression, macrophage infiltration and NF-κB signaling pathways [29, 30]. Other components in YQQRG, including *Lonicera japonica* [31], *Forsythia suspense* [32, 33], Duchesnea [34] and *Oldenlandia diffusa* [35] exhibit potential anti-inflammatory activities. The anti-apoptotic effects of astragalosides have been addressed by other researchers [36, 37], and administration of astragalosides may inhibit high glucose-induced renal tubular epithelial cell apoptosis [38]. Chlorogenic acid, the main component of *Lonicera japonica*, may protect cells against apoptosis by attenuating caspase-3 activation [39]. Luteolin in *Lonicera japonica* may inhibit the caspase-3 expression of tubular cells to ameliorate cisplatin-induced nephrotoxicity [40]. Leonurine in *Leonurus japonicus* may inhibit apoptosis through the mitochondria pathway [41]. Anemonin in *Poria cocos* appears to significantly decrease TUNEL-positive cells in rats with ischemia and reperfusion injury [36]. Nevertheless, as YQQRG is comprised of several herbal medicines and the actions of a single compound may be very different when combined in the formula, further studies are necessary to investigate the effects of YQQRG as a whole.

## Conclusions

The present study demonstrates that YQQRG might be a novel therapeutic agent in the treatment of nephrosis. Ameliorating renal inflammation and protecting resident renal cells from apoptosis is a possible underlying mechanism by which YQQRG inhibits PAN-associated renal injury.
